# One of the Lost Arts

**Published:** 1875-06-01

**Authors:** 


### "ONE OF THE LOST ARTS."

THIS is the title of a lengthy paper on blood-letting, read in one of the general sessions of the American Medical Association, during the recent meeting in Louisville, by Prof. S. D. Gross, of Philadelphia. The reading of the paper was listened to with much interest by a large audience, and most of the positions taken by the writer were undoubtedly correct. Yet we regretted to notice what appeared to us a vein of exaggeration in many of the expressions used, and sometimes, though rarely, a fault in the reasoning. It is probable that no one thing has contributed more to create general skepticism concerning everything pertaining to practical medicine than the apparently opposite or contradictory doctrines and modes of practice instituted by different parties and at different periods of time. Hence, it is not desirable, on any occasion, to exaggerate such contradictions. Neither is it quite just, or historically correct, to present the views and practices of extreme men, or men of extraordinary enthusiasm, as true representations of the generation to which they belong. When the author of the paper on "One of the Lost Arts" represents Dr. Rush and his followers as bleeding "in every possible disorder, until, in many cases, no more blood would flow because there was *none left*," to illustrate the indiscriminate use of the lancet at a former period, and then, in reference to the present, declares that "the lancet is an obsolete instrument, * * * and few of the present generation of medical men would, if called upon, be able to open a vein in a scientific and creditable manner," he certainly conveys to the public mind an exaggerated idea of the contrast presented by the two periods. This same tendency to extravagant expressions is seen in his comments on the pernicious effects of *authority* and *fashion* in medicine. Take the following as a specimen: "For nearly a third of a century the doctrine of a change of type in disease has tyrannized over the minds of medical men, and exerted a controlling influence on their practice. Of all these delusions, the latter, often called Toddism, after Dr. Todd, its author, has exercised the most pernicious and baneful effects upon civilized society. Ensconcing itself behind a false position, it has literally enslaved the medical world, entrapping alike the wise and the foolish, and sweeping over human life with a force equal to that of the fiercest and most destructive hurricane." As a specimen of faulty reasoning or induction, take the following: "If the modern practice be right, then the old practice must have been wrong, *deadly wrong*." Does the conclusion really follow the premise? May we not, in medicine, as in many other things, reach the same results by widely different means? May not two physicians, both well versed in the pathology of disease, and the *modus operandi* of medicine, treat the same disease with nearly equal success by remedial agents of entirely different composition and modes of action? We think few intelligent and thoughtful practitioners will venture to answer these questions in the negative. And if, instead of assuming that different modes of treating disease necessarily implied contradiction and "deadly wrong," we should review more carefully the *modus operandi* of each in its relations to the known laws of physiological and pathological action, it is more than probable that they would be found to be only different methods of reaching the same end; and instead of still further destroying the public confidence by holding up the history of medicine as a volume of self-stultifying contradictions, we would be able to reconcile many of its apparent differences, and arrive at a much more just appreciation of the actual merits of different modes of practice. We do not make these comments from any opposition to blood-letting. For with us the lancet has never become "obsolete." It has had its place in our pocket for forty years, and is still used sufficiently often to see that it is in good order. On the *modus operandi* of blood-letting, and its advantage over mere sedatives, we commend the following extract from the paper of Prof. Gross:

"Before I proceed to speak of local bleeding, let us briefly inquire into the mode of action of venesection, or, in other words, how the removal of blood from the system affords relief in inflammatory affections? This question can be easily answered. In the first place, the abstraction acts spoliatively, diminishing, as the name implies, the quantity of blood both in the part and system. Secondly, it weakens the power of the heart, and thereby prevents it from sending the blood with the same force and velocity into the suffering structures. Thirdly, it unlocks all the emunctories, and thus promotes secretion. Fourthly, it disgorges the vessels at the seat of the disease, restores the circulation, and places the absorbent vessels in a better condition for the removal of effused matter. And, last but not least, it favors the action of other remedies, as purgatives, diaphoretics, diuretics and anodynes.

"But it will be said that all these effects may and can readily be induced by the agency of other remedies, as aconite, veratrum viride, digitalis, mercury, and tartar emetic, and that, too, at much less cost to the system. That these articles are powerful depressants, lowering the heart's action and promoting secretion, no one at all acquainted with their virtues will question; but I deny that they exercise the same beneficial impression upon the vessels at the seat of the inflammation. When blood is drawn freely from a large vein at the bend of the arm, from a large orifice, to an approach to syncope, the vessels at the seat of the morbid action are unloaded, often to such an extent that the affected structures do not exhibit any marked difference in color from those in their immediate vicinity. Thus, for example, in violent conjunctivitis, the mucous membrane, the seat of the disease, always under such circumstances presents a perfectly blanched appearance, however red and engorged it may have been the moment before. Now, what occurs in the eye, in such a case, may reasonably be supposed to take place in any other part of the body when a patient is bled to a similar extent. In pleurisy, one of the immediate effects of the copious extraction of blood is a mitigation of the torturing pain which forms so prominent a symptom in this disease, due evidently to the diminished calibre of the vessels in the pleura, previously in a state of complete repletion. Has any one ever witnessed such an effect from the use of aconite, digitalis, veratrum viride, or tartar emetic? Never. No matter how these articles may be administered, whether singly or variously combined, they are simply depressants, not depressants and evacuants, as the abstraction of blood from a vein or an artery. There is no blanching of tissue from their use, no unloading of distended and crippled vessels, indeed, no direct appreciable effect of any kind.

"The more recent researches in pathological histology furnish a hint not easy to be mistaken as to the most salient treatment of inflammation in its earlier stages. The leading indication is to restore the paralyzed capillaries to their normal tonicity, so as to prevent structural changes in their walls, and facilitate the outward passage of the white globules with which they are choked. It is now well known that in every inflamed area there is marked hyperæmic distention of the blood-vessels, which are often crowded to their utmost capacity with leucocytes, which emigrate through the vascular walls, and, in conjunction with the effused blood-liquor, constitute the most important elements in inflammatory deposits. Hence, the object of treatment should be to restore the capillaries to their normal calibre, through the artificial induction of contraction of their walls, an effect which can be brought about, as is daily witnessed in many of the external inflammations, by cold applications, which, as is well ascertained, produce reflex contraction of the vessels. In inflammation of the more deeply-seated organs and tissues, however, this object can only be attained by spoliative bleeding, whereby the affected capillaries are relieved of their contents. In this way only can their tonicity be restored, the further effusion or migration of cell elements restrained, and the absorption of existing deposits favored.

"Another effect of bleeding, not to be overlooked in this discussion, is the diminution which it causes in the quantity of fibrine and white globules, so remarkably augmented in inflammatory affections. This change, of which I have witnessed many examples, was beautifully illustrated in the case of a young man, nineteen years of age, whom I attended, along with the late Dr. Charles Woodward, of Cincinnati, on account of a severe attack of pleurisy. Blood was drawn on three consecutive days, the first bleeding being performed about thirty-six hours from the commencement of the attack. The fluid, amounting to nearly a quart, was not only greatly buffed, but cupped on both sides of the crassamentum, as is shown in the specimen which is still in my possession. At the second operation, the fluid was buffed but not cupped; and at the third it was merely a little sizy, all pain and active inflammation having by this time disappeared. If such effects follow the use of the articles above mentioned, I am uninformed of the fact."

